# Varied spectrum of clinical presentation and mortality in a prospective registry of visceral leishmaniasis in a low endemicity area of Northern Italy

**DOI:** 10.1186/1471-2334-13-248

**Published:** 2013-05-30

**Authors:** Giovanni Cenderello, Ambra Pasa, Andrea Dusi, Chiara Dentone, Federica Toscanini, Nicoletta Bobbio, Elisabetta Bondi, Valerio Del Bono, Manuela Izzo, Giovanni Riccio, Marco Anselmo, Raffaella Giacchino, Maria Grazia Marazzi, Gabriella Pagano, Giovanni Cassola, Claudio Viscoli, Giuseppe Ferrea, Andrea De Maria

**Affiliations:** 1 S.C. Malattie Infettive Ente Ospedaliero Ospedali Galliera, Genova, Italy; 2 S.C. Oncologia Ente Ospedaliero Ospedali Galliera, Genova, Italy; 3 SSD Microbiologia ASL1 Imperiese-Ospedale Sanremo, Sanremo (IM), Italy; 4 SC. Malattie Infettive, ASL1 Imperiese-Ospedale Sanremo, Sanremo (IM), Italy; 5 SC Malattie Infettive Ospedale San Paolo ASL2, Savona, Italy; 6 Dipartimento di Scienze della Salute (DISSAL), University of Genova, Genova, Italy; 7 Istituto G. Gaslini, Genova, Italy; 8 Clinica Malattie Infettive, IRCCS AOU San Martino-IST Genova, Genova, Italy; 9 SC Malattie infettive Ospedale Santa Maria della Misericordia Albenga ASL-2, Albenga (SV), Italy; 10 S.S.Infettivologia, IRCCS AOU San Martino-IST Genova, Genova, Italy; 11 A.D.M, University of Genova, Largo R.Benzi 10, Genova, 16132, Italy

**Keywords:** Visceral leishmaniasis, Immunocompromised host, HIV

## Abstract

**Background:**

Visceral Leishmaniasis (VL) is endemic in 88 countries, in areas of relatively low incidence with a relevant proportion of immune suppressed patients clinical presentation, diagnosis and management may present difficulties and pitfalls.

**Methods:**

Demographic data, clinical, laboratory features and therapeutic findings were recorded in patients identified by a regional VL disease registry from January 2007 to December 2010.

**Results:**

A total of 55 patients (36 adults mean age 48.7 years, 19 children median age 37.5 months) were observed presenting with 65 episodes. All childen were immunocompetent, whereas adults affected by VL included both immunocompetent (n°17) and immunesuppressed (n°19) patients. The clinical presentation was homogeneous in children with predominance of fever and hepato-splenomegaly. A wider spectrum of clinical presentations was observed in immunocompromised adults. Bone marrow detection of intracellular parasites (Giemsa staining) and serology (IFAT) were the most frequently used diagnostic tools. In addition, detection of urinary antigen was used in adult patients with good specificity (90%). Liposomal amphotericin B was the most frequently prescribed first line drug (98.2% of cases) with 100% clinical cure. VL relapses (n°10) represented a crucial finding: they occurred only in adult patients, mainly in immunocompromised patients (40% of HIV, 22% of non-HIV immunocompromised patients, 5,9% of immunocompetent patients). Furthermore, three deaths with VL were reported, all occurring in relapsing immunocompromised patients accounting for a still high overall mortality in this group (15.8%).

**Conclusions:**

The wide spectrum of clinical presentation in immunesuppresed patients and high recurrence rates still represent a clinical challenge accounting for high mortality. Early clinical identification and satisfactory treatment performance with liposomal amphotericin B are confirmed in areas with low-level endemicity and good clinical standards. VL needs continuing attention in endemic areas where increasing numbers of immunocompromised patients at risk are dwelling.

## Background

Leishmaniasis is a complex of mammalian diseases caused by protozoans classified as *Leishmania* species (Order:kinetoplastida Family:trypanosomatidae) [1,2]. Natural transmission may be zoonotic or anthroponotic, and occurs through the bite of a phlebotomine sandfly species (order Diptera) of the genus *Phlebotomus* (old world) and *Lutzomyia* (new world) [3,4] with the domestic dog as the main reservoir for visceral disease in the Mediterranean basin but in other hyperendemic areas (or instance Sudan or India) humans act as the main reservoir.

Leishmaniasis is endemic to 88 countries, including Europe. In southern Europe the majority of reported cases are due to zoonotic visceral leishmaniasis (VL), which is fatal when untreated. The more benign cutaneous leishmaniasis (CL) is also present in this area. Incidence of leishmaniasis in humans is relatively low, ranging from 0.2 × 10^-6^ to 0.49 × 10^-6^ (85.3 × 10^-6^ including Turkey). This estimate corresponds to 700 reported new cases per year in Southern Europe (3,950/yr if Turkey is included). The disease is spreading north because of several risk factors, climate being only one. Changes to the environment, population movements, dog and vector mobility may contribute to the spread.

In the Netherlands Leishmania has been introduced by dogs returning from military missions, albeit without transmission due to lack of vector [5]. A pattern of northward spread and notification of autochtonous VL due to vector diffusion has been recorded in northern Italy [6,7] and southern Germany [8,9].

In addition to this developing epidemiological pattern, new clinical presentations have been recently described in developed areas including isolated lymphoadenopathy sustained by Leishmania infantum [10,11] or active chronic visceral leishmaniasis [12]. In this context, increasing numbers of patients with secondary immunodeficiency due to medication including immunosuppressive treatment, solid organ or bone marrow transplantation and chemotherapy in endemic areas may determine an increase in receptive hosts and may represent diagnostic and management difficulties [13].

To address these issues, a Regional Disease Register was established encompassing all Infectious Disease Units in a coastal area in Northern Italy. The Register has the primary aim of monitoring prospectively the diagnostic, clinical and therapeutic approach to VL in the region, and to identify possible critical areas where changes in perception and management might be required.

Aim of the present analysis was to evaluate and report on clinical presentation, diagnostic tools and treatment in patients identified during the first 4 years of activity.

## Methods

### Registry

The Ligurian Registry for Visceral Leishmaniasis (Registro Ligure Leishmaniosi viscerale, RiLLeVi) was established at the end of 2006 and began to record data from January 1st 2007. It is composed of 7 Infectious Diseases Hospital units in the administrative region Liguria (approx.1.5 M inhabitants). The Infectious Diseases (ID) Units are located in Sanremo, Pietra Ligure, Savona, Genova (3 Units), and La Spezia. Patients are referred from local Accidents and Emergency (A&E) departments, general practitioners or as outpatients. Workup as inpatients is recorded and collected for those with a diagnosis of VL. Twice a year, locally collected data are transmitted to the coordinators, and placed on a database. History, clinical evaluation diagnostic procedures and treatment schedules are collected. Cases and results are examined periodically and discussed during an annual meeting.

Each center independently cared for patients according to optimized baseline standards for the diagnosis and management of infectious diseases.

Ethical Committee approval was sought and obtained by the coordinating center for the observational data gathering. All patients provided informed consent for data gathering.

### Diagnostic procedures

Diagnostic criteria for leishmaniasis fulfilled WHO criteria for both clinical and laboratory definition in all the patients including immunocompetent adults, immunodeficient adults and children (main symptoms and positive parasitology and serology) [14].

Specific tests for the diagnosis of Leishmania infection included: microscopic demonstration of Leishmania amastigotes on Giemsa stained smears of bone marrow aspirates, serology by immunofluorescence (IFAT, Biomerieux, France) and urinary antigens (Katex, Kalon Diagnostics UK). Polymerase chain reaction (PCR) for the identification in blood or bone marrow of Leishmania DNA sequences in the kinetoplast region was performed as described elsewhere [15].

IFAT and Urinary antigen were performed at the Sanremo center. Cut-off values for positive IFAT results were 1:80 serum dilutions.

### Patient data, clinical presentation and treatment

According to registry requirements, epidemiological clinical and laboratory data were recorded for each patient each time an episode of VL was identified, including sex, age, town of residence, HIV infection, neoplasia or administration of immunosuppressive regimens, full haematological assessment, total protein, protein electrophoresis. Clinical evaluation of hepato- and splenomegaly were defined and graded according to clinical guidelines [16], anemia [17], neutropenia (<1500/μl) [18] and thrombocytopenia (<150,000/μl) [19] were defined as described. In HIV infected patients peripheral CD4+ T-cell counts, HIV viral load and drug history were recorded.

The main presenting aspects of the first clinical episode that led to VL diagnosis after admission to the ID unit was characterized and described in order to allow for possible heterogeneity of underlying clinical conditions and different patient age.

Treatment drugs, regimens, administration schedules and adverse events of grade 2 or higher associated with treatment were recorded.

No distinction between VL relapse and reinfection was possible. Patients presenting with recurrent episodes were therefore defined as “recurring” or “relapse” without distinction between relapse and reinfection.

### Statistical analysis

Categorical data were summarized as number and percentage of subjects, continuous data as mean, standard deviation and range. Comparisons between laboratory values were performed with the Kruskal-Wallis test for independent samples. Mann–Whitney, Wilcoxon (Breslow) and Chi-square tests were applied where needed. The time to relapse were evaluated with Log-rank test for equality of survivor functions and Wilcoxon test for equality of survivor functions. Statistical significance was defined as a P-value of less than 0.05 (two-sided). All statistical analyses were performed using the STATA 11.0 Software (StataCorp LP, College Station, TX) and SPSS 13,0(SPSS Inc, Chicago, IL). Statistical significance was defined as a P-value of less than 0.05.

## Results

### Epidemiology of identified cases

**Figure 1:**
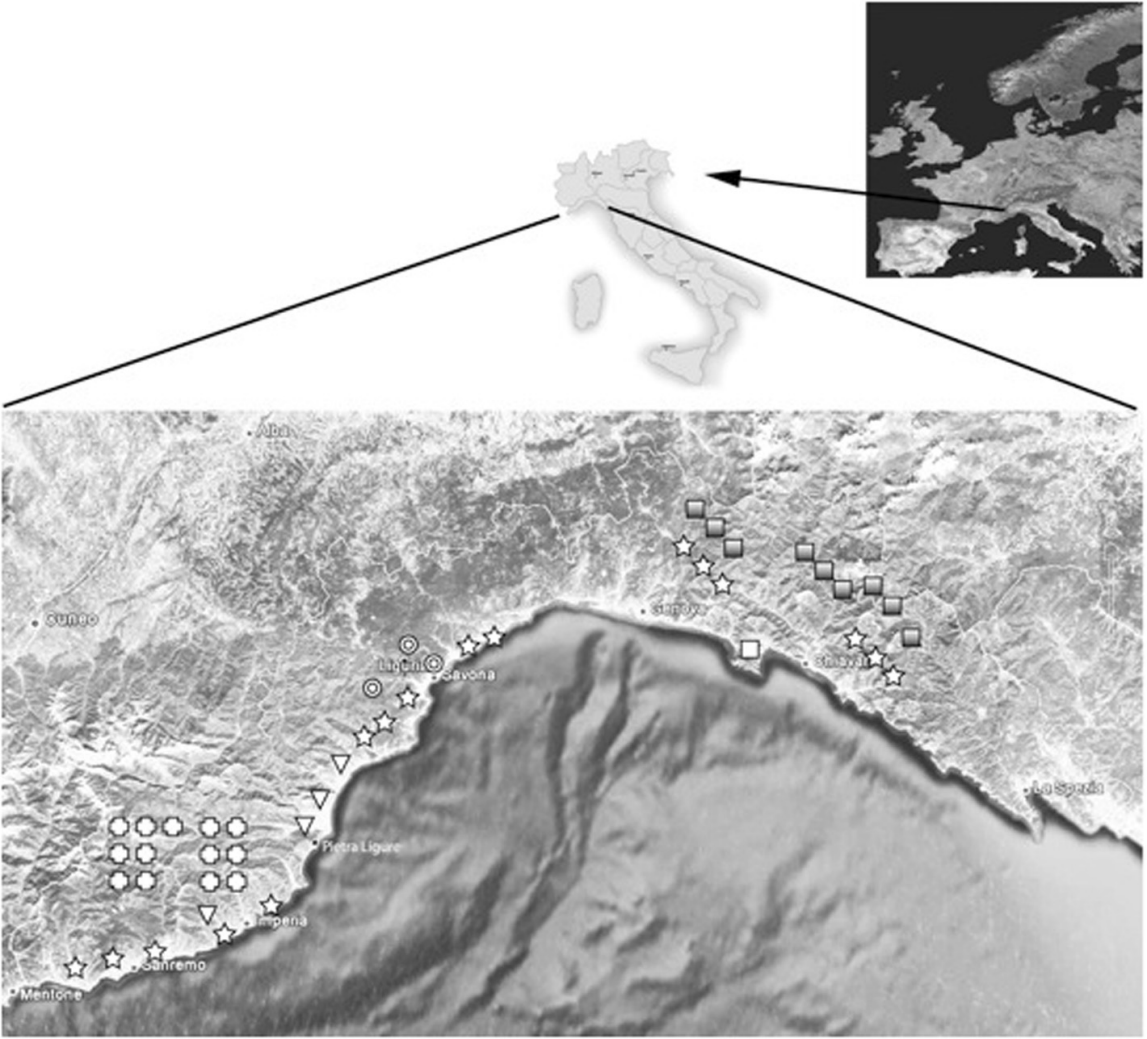
**Map showing area of residency of patients in Liguria region and location within Italy and Europe. **Signs show area of residency and hospital Infectious Diseases Unit where seen as inpatients. Star: children seen at Gaslini Hospital, Cross: patients seen at Sanremo Unit, Triangles: patients seen at Pietra Ligure Unit, Circles: patients seen at the Savona Unit, Greyed Squares: Patients seen at the Galliera Hospital Unit, Open Square: patients seen at the IRCCS S. Martino Hospital Unit.

Information on patients with confirmed VL diagnosis was collected from 01/01/2007 to 31/12/2010. All the seven Infectious diseases Units from the region were involved and participated. Five units actively reported confirmed VL cases. Of five centers one is in a tertiary care pediatric Institute (G.Gaslini) and reported exclusively pediatric cases. Two units did not observe VL cases in their area. Figure 1 indicates the area of origin where patients were living.

Sixty-five episodes of VL were recorded in 55 patients (36 adults, 19 children) (Table 1). Up to three VL relapses occurred in 7 (12,5%) adult patients requiring additional evaluation and treatment as inpatients.

**Table 1:** VL cases, first episodes and recurrences

	**Cause for immunocompromise**	**Patients**	**Total episodes**	**Recurring episodes**
Immunocompetent		17	18	1
Immunocompromised		19	28	9
	HIV	10	15	5
	non-HIV	9	13	4
**Subtotal**		**36**	**46**	**10**
Children		19	19	0
**Total**		**55**	**65**	**10**

The mean age of pediatric patients (7 M, 12 F) was 37,5 months (range:11–155), and adult patients (21 M, 15 F) had a mean age of 48,7 yrs (range 21–87). All patients stably lived in the Liguria coastal area, with the exception of three children being referred from other Italian endemic areas.

All the children were immunocompetent, as defined by absence of primary immunodeficiency, autoimmunity, cancer, HIV infection or previous administration of potentially immunosuppressive treatment. The 36 adult patients were subdivided into two groups according to their immune status. Of these, 17 (47%) were immunocompetent (ICC), and 19 (53%) were immunocompromised (ISS).

HIV-1 infection was the dominant cause of immunosuppression (10 pts, 59%), while the other 9 patients (41%) had variable underlying diseases requiring immunosuppressive treatment including steroid therapy (6pts), chemotherapy in multiple myeloma, rituximab chemotherapy for Non Hodgkin Lymphoma and cyclophosphamide-steroid treatment in a patient with pseudohemophilia and anti-factor VIII Ab (1 case each). Multiple episodes of VL were recorded in some patients over the study period. Therefore, the present evaluation takes into account VL episodes where indicated.

### Clinical features and presentation

Overall clinical features recorded among all patients are summarized in Table 2. The main presenting clinical picture, defined as the clinical/laboratory values that led to referral to the Infecitous Diseases Units are indicated in Table 3. All the 19 children with VL were immunocompetent as opposed to adults, who had an underlying cause of immunosuppression in 52% (19/36) of the instances (Chi square, p = 0.001). Children were all febrile at presentation, while 4/36 (10%) of adults were afebrile and reported no fever in preceding days. The most frequently observed clinical presentation was fever associated with liver and spleen enlargement. This presentation was more frequent in children (100%) compared to adults (54.3%), (Chi-square p = 0.001).

**Table 2:** Symptoms and blood laboratory data in patients with VL, stratified according to risk group

	**Children (19pts)**	**ICC (17pts)**	**ISS HIV+ (10pts)**	**ISS HIV-(9pts)**
Constitutional Symptoms	19(100%)	15 (88.2%)	10 (100%)	9 (100%)
Hepato + Splenomegaly	19 (100)	11 (65%)	8 (8%)	6 (66%)
Lymphoadenopathy	0	1 (6%)	0	0
Other symptoms	0	0	2 (20%)	1 (1%)
Haematological data	18 (95%)	17(100%)	10(100%)	9(100%)
--Anemia	12(63.2%)	10(58.8%)	10(100%)	6(66.6%)
--Leukopenia	15(78.9%)	8(47.1%)	8(80%)	0
--Trombocytopenia		2(11.8%)	1(10%)	0
--Pancytopenia		0	6(60%)	2(33.3%)
-Hypergammaglobulinemia	15((78.9%)	5(29.4%)	8(80%)	6(66.6%)

**Table 3:** Clinical presentation on admission according to patient risk group

**Patient group**	**F + S**	**F + L**	**L**	**F + P**	**F + H**	**T**	**Other**
Children (19)	19 (100%)	0	0	0	0	0	0
Adults (46)	25 (54,3%)	2 (4,3%)	2 (4,3%)	8 (17,4%)	4 (8,7%)	2 (4,3%)	3 (6,5%)
ICC(18)	11 (61,1%)	0	2	0	4	0	1
IDD (28)	14 (50%)	2 (7,1%)	0	8 (28,6%)	0	2 (7,1%)	2 (7,1%)
HIV + (15)	8 (53,3%)	2 (13,3%)	0	2 (13,3%)	0	2 (13,3%)	1 (6,6%)
non-HIV (13)	6 (46,15%)	0	0	6 (46,15%)	0	0	1 (6,7%)

Other: Gastritis, and Haemophagocytic syndrome, 1 presentation each.

*F *fever, *S *splenomegaly, *L *lymphadenopathy, *P* pancytopenia, *H *hyper-gammaglobulinemia, *T *thrombocytopenia.

Among the 28 VL episodes in immunocompromised patients, fever (>37,5°C) and peripheral cytopenia (8 pts) was the second most frequently recorded clinical presentation after fever and hepato- splenomegaly, and was unique to immunocompromised compared to immunocompetent patients (8/28pts vs.0/28 pts, respectively, Chi-square p = 0,003). Fever and hypergammaglobulinemia were recorded on admission only in adult immunocompetent patients, while reduced cytopenia was observed only in immunocompromised adults.

When considering haemoglobin levels, significant differences emerged among the three patient groups (9.69 ± 1.88, 10.11 ± 2.28, 8.02 ± 0.90 g/dl) in immunocompromised adults, immunocompetent adults, children respectively). Overall, mean Hb concentration was 8.85 gr/dl (6,60-15 gr/dl) with significant differences and lowest mean values in children, (p = 0.001, Kruskall-Wallis).

Average white blood cell (2661 ± 952 3842 ± 3122, 4353 ± 2170/μl, in immunocompromised adults, immunocompetent adults, children respectively) and neutrophil counts (1766 ± 767 2262 ± 2420, 1091 ± 645/μl in immunocompromised adults, immunocompetent adults, children respectively) were below the lower laboratory reference limits in all patient groups. Significant differences were present for both leukocyte (p = 0.032 Kruskall-Wallis) and neutrophil counts (p = 0.017, Kruskall-Wallis), with children displaying lowest neutrophil counts, and immunocompromised patients lowest leukocyte counts.

In addition, mean total blood protein (7.39 ± 1.24, 7.76 ± 1.16, 7.68 ± 0.92 g/dl, in immunocompromised adults, immunocompetent adults, children respectively) and albumin (2.88 ± 0.68 3.28 ± 0.60 2.98 ± 0.56 g/dl, in immunocompromised adults, immunocompetent adults, children respectively) values were similar among the three groups of patients, while γ-globulin concentrations (3.00 ± 1.02, 2.46 ± 1.12, 1.72 ± 0.49 g/dl in immunocompromised adults, immunocompetent adults, children respectively) were consistently different in adults and children with lowest values in pediatric patients (p < 0,0001, Kruskall-Wallis).

### VL diagnostic methods

Among the tools used for VL diagnosis, Giemsa staining and microscopic examination of bone marrow samples was the most frequently used technique (48/65 episodes, 73%). Microscopic examination of bone marrow aspirates was more frequently employed in children (18/19 episodes, 94,7%) compared to adults (30/46 episodes, 62,1%) Among adults, bone marrow aspiration was performed more frequently in VL episodes in ICC (15/18) than in ISS (16/28). No differences in diagnostic procedure employment were statistically significant.

Table 4 reports for each patient group the comparative methods employed and its performance expressed as the proportion of patients with a diagnosis of Leihmaniosis at first observation correctly identified by a given diagnostic procedure. The identification of anti-Leishmania serum antibodies by immunofluorescence (IFAT) is a reliable, non-invasive diagnostic tool. Here, serology for Leishmania was carried out in 60/65 VL episodes (92,3%). Overall, anti-Leishmania antibodies were detected in 14/15 (93%) of episodes occurring in children and in 43 of 45 (95,6%) VL episodes recorded among adults with similar rates of positivity in immunocompetent and immunocompromised patients.

**Table 4:** Relative performance of Diagnostic tests for Leishmaniasis

	**I.F. Serology**			**Microscopy (BM)**			**Urinary Ag**			**PCR - P. Blood**			**PCR-Bone Marrow**		
***Group (n°)***	***POS***	***NEG***	***ND***	***POS***	***NEG***	***ND***	***POS***	***NEG***	***ND***	***POS***	***NEG***	***ND***	***POS***	***NEG***	***ND***
Children(19)	**14** (73,6%)	**1** (5.3%)	**4**	**17** (89.5%)	**1** (5.3%)	**1**	0	0	**19**	**1** (5.3%)	0	**18**	**8** (42.1%)	0	**11**
Adults(36)	33(91,7%)	2(5,6%)	1	19(52,8%)	4(11,1%)	13	25(69,5%)	3(8.3%)	8	5(13,9%)	2(5,6%)	29	3(8,4%)	0	33
ICC(17)	15(88,2%)	1(6,7%)	1	11(64,7%)	4(23,5%)	2	8(47.0%)	2(11,8%)	7	3(17,6%)	0	14	0	0	17
ISS(19)	18(94,7%)	1(5,3%)	0	9(47,4%)	2(10,5%)	8	10(52,6%)	1(5,3%)	8	2(10,6%)	2(10,6%)	15	3(15,8%)	0	16
HIV(10)	10(100%)	0	0	3(30%)	1(10.0%)	6	7(70.0%)	0	3	2(20.0%)	1(10.0%)	7	2(20.0%)	0	8
Non-HIV(9)	8(88,9%)	1(11,1%)	0	6(66,7%)	1(11,1%)	2	3(33,3%)	1(11,1%)	5	0	1(11,1%)	8	1(11,1%)	0	8

Numbers in brackets refer to the proportion (%) of patients actually evaluated by a given diagnostic method. *ICC *immunocompetent, *ISS *immunocompromised, *HIV *HIV infected. HIV and non-HIV are subgroups of the ISS patients.

Identification of Leishmania urinary antigen was centralized, introduced as a diagnostic support and offered to the other centers within the registry for suspected cases. Overall, it was employed in 30/46 (65%) adult episodes, (55.6% of immunocompetent and 71,4% of immunocompromised adults) while none of the children was tested for the presence of urinary antigens. Urinary Leishmania antigen was detected in 27/30 (90%) of episodes (8/10(80%) in Immunocompetent and 19/20 (95%) in immunesuppresed patients.

Polymerase chain reaction (PCR) for Leishmania-specific sequences in bone marrow aspirates was performed at a National Reference Laboratory (Dr. L. Gradoni, Istituto Superiore di Sanità, Rome) in 11 patients with 100% positive results, and on peripheral blood of 9 additional patients with positive results in 6 cases (66,7%) [15].

In children, microscopic detection on bone marrow samples was positive in 15/18 (83,3%).

### Treatment courses and VL relapse episodes

Liposomal amphotericin is the drug of choice for the treatment of VL in Europe [20-22]. Indeed, during the observation period of the registry 62 (95%) episodes of VL were treated with liposomal amphotericin B with amphotericin B lipid emulsion being used in one instance. Miltefosine was administered to 2 patients during a VL relapse episode.

Overall, there was a good homogeneity of prescription schedules. Three different schedules of liposomal amphotericin B administration were recorded: 3 mg/kg “short” regimen in children and ICC adults (days 1➔ 5,day 14,day 21;36 episodes), a 3 mg/kg “extended” regimen in ISS adults (days 1➔5,10,17,24,31,38; 10 episodes) and a 4 mg/kg “extended” regimen in 16 episodes of VL occurring in ISS adults. Amphotericin B lipid emulsion was prescribed at 3 mg/kg according to a “short” regimen. Miltefosine was administered at 2.5 mg/kg according to registration studies [23] for successful full 28 days courses in two immunocompromised adults following clinical failure on liposomal Amphotericin B or VL relapse.

**Figure 2:**
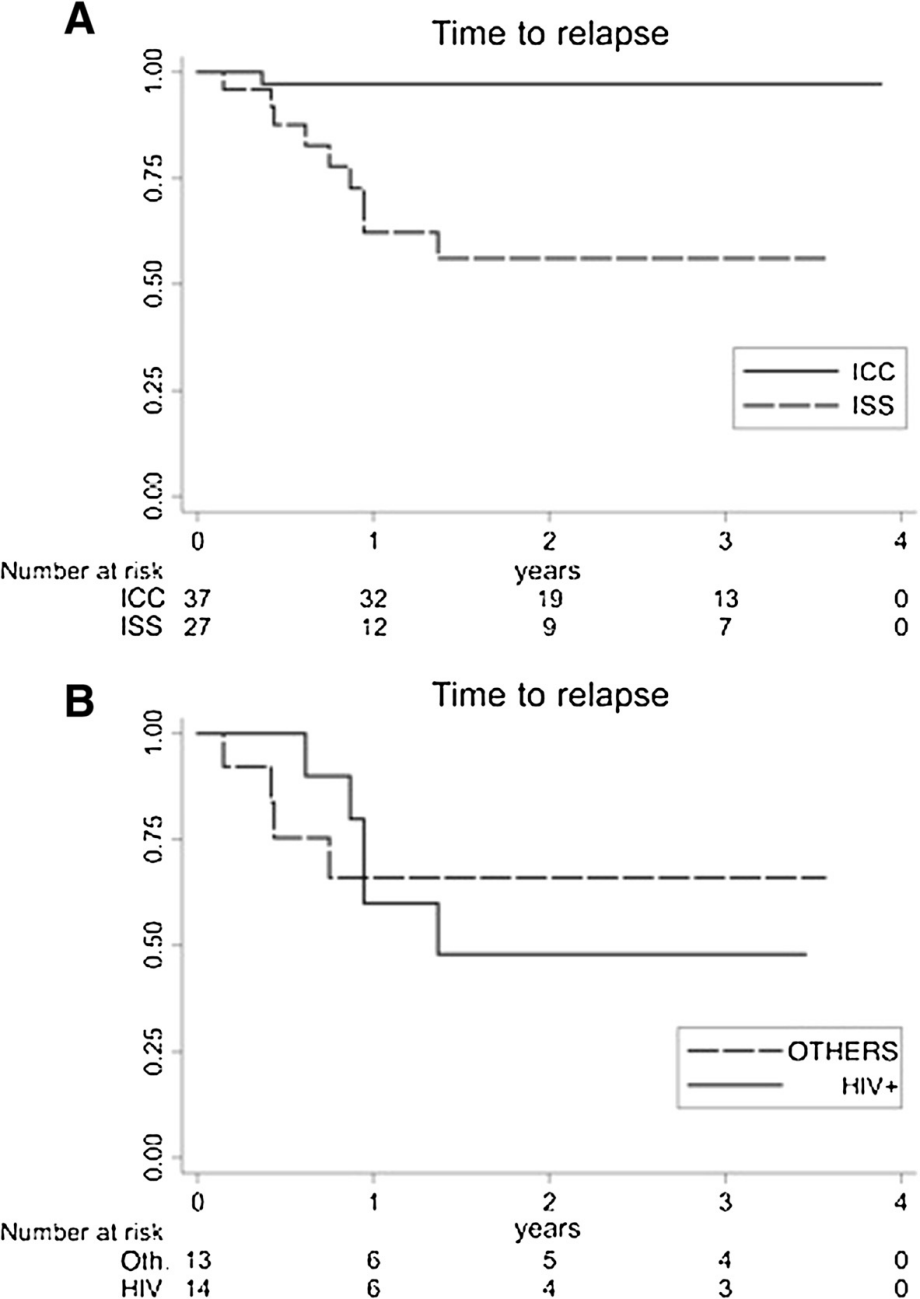
**Kaplan-Meyer analysis of Relapse-free time after the first Visceral Leishmaniasis event among observed patients. **Panel **A**. Time to relapse (in days) in Immunocompetent(ICC)vs. Immunocompromised (ISS) pts. Log-rank test for equality of survivor functions (chi2 = 0.0002); Wilcoxon (Breslow) test for equality of survivor functions (Pr > chi2 =0.0004). Panel **B**. Time to relapse (in days) in Immunesuppresed pts.: HIV + vs. other causes (Others). Log-rank test for equality of survivor functions (chi2 = n.s.); Wilcoxon (Breslow) test for equality of survivor functions (Pr > chi2 = n.s.).

When analyzing VL recurrent episodes, these were observed in 5/10 (40%) HIV+, 4/9 (22.2%) non-HIV immunocompromised and 1/17 (5,9%) immunocompetent patients. Ten episodes of VL recurrence were registered among 46 episodes diagnosed in infected adult patients (21,7%). No VL relapse was observed during follow up of the children. Of the 10 recurring episodes, 9 occurred in immunocompromised adults after a median of 331 days from the first VL episode, and one was observed in an otherwise immunocompetent adult after 143 days. This difference was significant by Kaplan-Meyer analysis (p < 0.0001) (Figure 2 panel A). Among VL recurring episodes, there was no difference in the frequency between HIV-infected and non-HIV infected immunocompromised patients (Figure 2 panel B), and time to recurrence was not significantly different (345 days vs. 314 days). Clinical features at relapse were similar to those detected at the first presentation for each patient with at least one recurrence of VL.

None of the immunocompromised patients subsequently presenting with a second VL episode were on secondary prophylaxis. Among HIV infected patients, mean CD4+ cell counts and viremia at first VL diagnosis were 135 ± 101/μl and 6,974 × 10^4^ cp/ml (range: 0–2,8 × 10^5^), respectively. In 3/10 patients CD4+ cell counts were >200 μl at diagnosis with 2 of these 3 cases recording CD4+ cells >250÷μl. No significant CD4+ cell count differences were present among those who would not recur compared to those for whom a VL recurrence was observed (148 ± 129/μl vs. 115 ± 40/μl, respectively) Among the 5 VL relapses in HIV infected patients, CD4+ cell counts at recurrence were 152 ± 89,39/μl (Mean ± DS). CD4 cell numbers at VL recurrence were not different compared to those for the patients at baseline (1st VL diagnosis). Among the 10 HIV patients with VL episodes, 4 were not on HAART and VL represented their first opportunistic infection, albeit non AIDS-defining. Among the other 6 patients a failing HAART regimen with persistent viremia >50 cp/ml was recorded in 4 cases.

Mortality in this cohort was assessed for all patients during the follow up period as any incident case of death within the cohort included in the registry during the observation period. Overall, 3 deaths were recorded among the 55 patients (5.5%). Patients who died were all immunocompromised (3 of 19, 15,8%). Two patients died with leishmaniasis but cause of death was attributed to disseminated Mycobacterium Avium-intracellulare Complex in 1 case and to complications of carcinoma of the lungs (1 patient). One patient died due to Leishmaniasis. Of these 3 patients (2 HIV+, 1 non-HIV), 2 had experienced VL relapse. Mortality was high among relapsing patients (2 of 5 relapsing HIV + patients, 1 of 4 relapsing non-HIV immunocompromised patients).

Thus, treatment regimens were quite homogeneous during the survey. A high rate of VL recurrence in adult patients was observed in both HIV-infected and in HIV-uninfected immunocompromised patients. High mortality rates are associated to VL relapse and poor clinical condition.

## Discussion

Visceral leishmaniasis is a neglected disease with frequent underdiagnosis and underreporting in both developing and developed areas [24,25]. Results of this registry/surveillance program points towards a changing spectrum of disease presentation in adult patients, and high rates of relapse in immunocompromised adults, both HIV-infected and HIV-uninfected. Immunocompromised patients represented 53% of adult patients with VL in this study. The clinical presentation in these patients was classical (i.e.: fever, hepatomegaly, splenomegaly) in only 50% of instances. The apparent change in disease spectrum was thus due to a shift in the composition of patient groups presenting with VL disease, with a high proportion of immunocompromised patients being notified to the registry. In addition, the relevant number of relapses observed may be regarded as a change from what we are used to observe or from what is usually described/expected. These features are likely to be increasingly observed with the persistence of Leishmania transmission in endemic areas, with its spread northwards (6,7) and with the increase in the prevalence of immunocompromised patients (both HIV + and HIV-) in these areas.

Of the 55 enrolled patients in the present study, 34% were otherwise immunocompetent HIV-uninfected children. VL clinical presentation features were quite homogeneous in pediatric patients and did not differ from those previously reported from the mediterranean basin in Italy [26] and southern France [27], with fever and hepatosplenomegaly being the main presenting complaint. Compared to those reported from Sicily [26] children in the present report were older (3 vs. 1.7 yrs) and had neither concomitant infections/diseases, nor cutaneous manifestations of visceral invasive disease. None of the children was diagnosed with advanced VL features including ascites or hemorrage, nor presented with diarrhea or bronchopneumonia as recently reported in a high proportion (27% and 84% respectively) of pediatric cases in a retrospective study in Albania [28]. These differences could reflect clinical alertness of the pediatric regional network and overall early referral to the main regional pediatric institute with high clinical standards. On the contrary, adult patients reported to the registry had a wide variability of clinical presentation. Significantly increased proportions of fever and bone marrow defects were observed in immunocompromised patients in the absence of hypergammaglobulinemia. This variable pattern of VL presentation in adult patients, together with the fragmentation of incident cases over different centers (see map), may represent a challenge for initial diagnostic workup in areas of low endemicity, higher prevalence of immunosupressed patients, or in areas where VL is presently spreading [6-8].

The development and use of non-invasive and robust diagnostic tools in addition to IFAT could be of help in the diagnosis of suspected VL cases without requiring invasive procedures including bone marrow or splenic aspirate. In the present work, urinary antigen detection was used exclusively in adult patients. In this setting, its sensitivity was good and compared well with serology (IFAT) both in immunocompetent and in immunesuppresed adults. This finding is in line and confirms previous data where specificity was 96% with sensitivity of 85.7 and 100% [29,30]. Thus, in areas of focal scattered incident cases with a mostly vector-borne low endemicity, or in developing areas of the world, the addition of leishmania urinary antigen detection to IFAT may avoid the need to adopt more costly or invasive techniques (i.e. leishmania PCR-DNA detection and bone marrow aspiration, respectively) that may be available only in referral centers. Another rapid serology test, the k39 dipstick test [31], has proven to be highly specicfic and sensitive for the diagnosis of leishmaniasis. Due to its unavailability for routine clinical use, we could not compare the performance of urinary antigen and k39 dipstick tests.

VL recurrence in HIV patients is a well characterized problem and the main clinical predictors of this event include: absence of a CD4+ increase at follow up, lack of secondary prophylaxis and CD4+ cell counts <100/μl at the time of primary VL diagnosis [32]. We here observed some differences compared to previous reports [33,34]. Most of our HIV coinfected patients were either off HAART or in a failing HAART regimen at VL diagnosis (8/10) and relapsers had either no rise in CD4+ cells or were not administered secondary prophylaxis. However, most of them had CD4+ cell counts well above 100/μl, and in 30% of the cases >200/μl. In addition, multiple relapses were recorded in a virologically suppressed patient, and one relapse was observed in a non-HIV immunocompetent patient. Thus, although HAART has radically changed the epidemiology and incidence of VL in HIV patients [35,36], in the presence of prolonged or failing HAART, other factors may be associated to the appearance of VL at higher CD4+ cell counts. HIV-Leishmania coinfection in these patients may reproduce a vicious cycle leading to persistent immune derangement and paradoxical persistence of low CD4+ cell counts and parasitemia despite HAART and liposomal amphotericin B administration [37,38]. In this context, the good sensitivity of urinary leishmania antigen detection observed in this study, could represent a useful tool to monitor potential VL relapse/persistence. Indeed, while monitoring post-treatment cure in a series of 130 patients, detection of urine parasite antigens was not associated with clinical disease in 54% of patients [29] and could represent clinical latency with parasite persistence. Its use for monitoring immunocompromised patients after completion of treatment could therefore provide additional support in the management of VL patients with secondary prophylaxis regimens, and help identify those at risk of relapse. In this context, also the k39 dipstick may identify persistent antigenemia that may be interpreted as asymptomatic infection [39]. The present work was limited by the impossibility to discriminate between Leishmania reinfection or relapse therefore the possibility of new infections among immunocompromised patients could not be ruled out. Given previous experience in this area [33,34], however, it is likely that the majority of relapses observed among immunocompromised patients were “true relapses” rather than reinfections.

Larger patient numbers and prospective studies may be suited to confirm the presently observed changing pattern and high mortality of patients with VL relapses due to the increasing prevalence of immunocompromised hosts and still incomplete control of VL through secondary prophylaxis.

## Conclusion

In conclusion the present data provide a picture of what is actually occurring in everyday clinical practice and may offer a “snapshot” on the often underreported or underestimated cases of VL relapse that may need more intensive clinical efforts at improving secondary VL prophylaxis, VL monitoring using leishmania antigen tests (urinary or serum), and HAART intensification to improve patient survival.

## Abbreviations

VL: Visceral leishmaniasis; RiLLeVi: Registro Ligure Leishmaniosai Visceral; ID: Infectious diseases unit; A&E: Accidents and emergency; IFAT: Immunofluorescence detection antibody; ICC: Immunocompetent patient; ISS: Immunesuppresed patient; HAART: Higly active antiretroviral therapy.

## Competing interests

The authors declare that they have no competing interests.

## Authors’ contributions

Conceived and designed the study: GC, ADM, GP. Performed the study all. Analyzed the data: AP, ADM, GC Contributed reagents/materials/analysis tools: AD. Wrote the paper: ADM, GC,CV. Critical review all. All authors read and approved the manuscript.

## Pre-publication history

The pre-publication history for this paper can be accessed here:

http://www.biomedcentral.com/1471-2334/13/248/prepub

## References

[B1] WHO Report on Global Surveillance of Epidemic-prone Infectious Diseases Chapter ten Leishmaniasis and Leishmania HIV/coinfection: Background Information 2009 WHO Available from: http://www.who.int/csr/resources/publications/surveillance/Leishmaniasis.pdf last accessed May 03rd 2012

[B2] Ready PD de la Roque S Leishmaniasis emergence and climate change Climate change: the impact on the epidemiology and control of animal diseases 2008 27 399 412 Rev Sci Tech Off Int Epiz

[B3] Kuillick-Kendrick R Phlebotomine vectors of the leishmaniases: a review Med Vet Entomol 1990 4 1 24 10.1111/j.1365-2915.1990.tb00255.x 2132963

[B4] Peters N Sacks D Immune privilege in site of chronic infection: leishmania and regulatory T cells Immunol Rev 2006 213 159 179 10.1111/j.1600-065X.2006.00432.x 16972903

[B5] Hovius E Pinelli E Nijsse R Poot J van der Giessen J Introduction of leishmania species in the Netherland from dogs who are returning from military missions and vacations in countries where leishmaniasis is endemic Tijdschr Diergeneeskd 2011 136 344 348 21614851

[B6] Maroli M Rossi L Baldelli R Capelli G Ferroglio E Genchi C Gramiccia M Mortarino M Pietrobelli M Gradoni L The northward spread of leishmaniasis in Italy: evidence from retrospective and ongoing study studies on the canine reservoir and phlebotomine vectors Trop Med Int Health 2008 13 256 264 10.1111/j.1365-3156.2007.01998.x 18304273

[B7] Biglino A Bolla C Concialdi E Trisciuoglio A Romano A Ferroglio E Asymptomatic Leishmania infantum infection in Area of Northwestern Italy (Piedmont Region) Where such infections are traditionally nonendemic J Clin Microbiol 2010 48 131 136 10.1128/JCM.00416-09 19923480 PMC2812267

[B8] Bogdan C Schönian G Bañuls AL Hide M Pratlong F Lorenz E Röllinghoff M Mertens R Visceral leishmaniasis in a child who had never entered a known endemic area: case report and review of the literarature Clin Inf Dis 2001 32 302 306 10.1086/318476 11170923

[B9] Harms G Schonian G Feldmeier H Leishmaniasis in Germany Emerging Infect Dis 2003 9 872 875 10.3201/eid0907.030023 12890332 PMC3023440

[B10] Kumar B Verma P Role of fine-needle aspiration cytology in the prompt diagnosis of recurrence of visceral leishmaniasis presented as isolated cervical leishmanial lymphadenopathy Diagn Cytopathol 2013 Feb 41 2 150 2 10.1002/dc.21747 21671412

[B11] Pomares-Estran C Cenderello G Ittel A Karsenti JM Cardot-Lçeccia N Vassallo M Hasseine L Delaunay P Rosenthal E Marty P Isolated lymphoadenopathy in Leishmania infantum infection:three case reports Ann Trop Med Parasitol 2009 103 555 559 10.1179/000349809X12459740922093 19695161

[B12] Bourgeois N Bastien P Reynes J Markinson A Rouanet I Active Chronic leishmaniasis in HIV-1 infected patients demonstrated by biological and clinical long term follow up of 10 patients HIV Med 2010 11 670 673 10.1111/j.1468-1293.2010.00846.x 20500233

[B13] Cascio A Iaria M Iaria C Leishmaniasis and biologic therapies for rheumatologic diseases Semin Arthritis Rheum 2010 40 e3 e5 10.1016/j.semarthrit.2009.07.001 19782386

[B14] WHO/PAHO Visceral Leishmaniasis WHO/PAHO Epidemiol Bull 2002 23 14 12608348

[B15] Oliva G Scalone A Foglia Manzillo V Gramiccia M Pagano A Di Muccio T Gradoni L Incidence and time course of Leishmania infantum infections examined by parasitological, serologic, and nested-PCR techniques in a cohort of naive dogs exposed to three consecutive transmission seasons J Clin Microbiol 2006 44 1318 1322 10.1128/JCM.44.4.1318-1322.2006 16597857 PMC1448675

[B16] Bergasa MV Goldman L, Ausiello D **Approach to the patient with liver disease (**2007) Cecil textbook of Medicine 2007 23 Philadelphia: Saunders Elsevier 1089 1090

[B17] World Health Organisation Nutritional anaemias report of a WHO Scientific Group World Health Organ Tech Rep Ser 1968 405 5 37 4975372

[B18] Hsieh MM Everhart JE Byrd Holt D Tisdale JF Rodgers GP Prevalence of neutropenia in the US Population: Age Sex, Smoking status, and ethnic differences Ann Inter Med 2007 146 486 492 10.7326/0003-4819-146-7-200704030-00004 17404350

[B19] Buckley MF James JW Brown DE Whyte GS Dean MG Chesterman CN Donald JA A novel approach to the assessment of variations in the human platelet count Thromb Haemost 2000 83 480 484 10744157

[B20] Lachaud L Bourgeois N Plourde M Leprophon P Bastien P Ouellette M Parasite susceptibility to amphotericin B in failures of treatment for visceral leishmaniasis in patients coinfected with HIV type1 and Leishmania infantum Clin Infect Dis 2009 48 e16 e22 10.1086/595710 19093811

[B21] Buffet PA Rosenthal É Gangneux JP Lightburne E Couppié P Morizot G Lachaud L Marty P Dedet JP Therapy of Leishmaniasis in France: consensus on proposed guidelines Presse Med 2011 40 173 184 10.1016/j.lpm.2010.09.023 21106333

[B22] Bern C Adler-Moore J Berenguer J Boelaert M den Boer M Davidson RN Figueras C Gradoni L Kafetzis DA Ritmeijer K Rosenthal E Royce C Russo R Sundar S Alvar J Liposomal Amphotericin B for the Treatment of Visceral Leishmaniasis Clin Infect Dis 2006 43 917 924 10.1086/507530 16941377

[B23] Sundar S Jha TK Thakur CP Engel J Sindermann H Fischer C Junge K Bryceson A Berman J Oral Miltefosine for Indian Visceral Leishmaniasis N Engl J Med 2002 347 1739 1746 10.1056/NEJMoa021556 12456849

[B24] Dujardin JC Campino L Cañavate C Dedet JP Gradoni L Soteriadou K Mazeris A Ozbel Y Boelaert M Spread of vector-borne diseases and neglect of Leishmaniasis Europe Emerg Infect Dis 2008 14 1013 1018 10.3201/eid1407.071589 18598618 PMC2600355

[B25] Chappuis F Sundar S Hailu A Ghalib H Rijal S Peeling RW Alvar J Boelaert M Visceral leishmaniasis: what are the needs for diagnosis, treatment and control? Nat Rev Microbio 2007 5 873 882 10.1038/nrmicro1748 17938629

[B26] Cascio A Colomba C Antinori S Orobello M Paterson D Titone L Pediatric visceral leishmaniasis in Western Sicily, Italy: a retrospective analysis of 111 cases Eur J Clin Microbiol Infect Dis 2002 21 277 282 10.1007/s10096-002-0707-3 12072938

[B27] Minodier P Piarroux R Garnier JM Unal D Perrimond H Dumon H Pediatric visceral leishmaniasis in southern France Pediatr Infect Dis J 1998 17 701 704 10.1097/00006454-199808000-00008 9726344

[B28] Petrela R Kuneshka L Foto E Zavalani F Gradoni L Pediatric Visceral Leishmaniasis in Albania: A retrospective analysis of 1210 Consecutive Hospitalized Patients (1995–2009) PLoS Negl Trop Dis 2010 4 e814 10.1371/journal.pntd.0000814 20838650 PMC2935397

[B29] Riera C Fisa R Lopez P Ribera E Carrió J Falcó V Molina I Gállego M Portús M Evaluation of a latex agglutination test (Katex) for detection of Leishmania antigen in urine of patients with HIV -Leishmania coinfection: value in diagnosis and post treatment follow up Eur J Clin Microbiol Infect Dis 2004 23 899 904 15599651 10.1007/s10096-004-1249-7

[B30] Vilaplana C Blanco S Domínguez J Giménez M Ausina V TUral C Muñoz C Non invasive method for diagnosis of visceral leishmaniasis by a latex agglutination test for detection of antigens in urine samples J Clin Microbiol 2004 42 1853 1854 10.1128/JCM.42.4.1853-1854.2004 15071070 PMC387579

[B31] Bern C Jha SN Joshi AB Thakur GD Bista MB Use of the recombinant K39dipstick test and the direct agglutination test in a setting endemic for visceral leishmaniasis in Nepal Am J Trop Med Hyg 2000 63 153 157 11388508 10.4269/ajtmh.2000.63.153

[B32] Cota GF De Sousa MR Rabello A Predictors of visceral leishmaniasis relapse in HIV-Infected patients: a Systematic review PLoS Negl Trop Dis 2011 5 e1153 10.1371/journal.pntd.0001153 21666786 PMC3110161

[B33] Pasquau F Ena J Sanchez R Cuadrado JM Amador C Flores J Benito C Redondo C Lacruz J Abril V Onofre J Leishmaniasis as an opportunistic infection in HIV-infected patients: determinants of relapse and mortality in a collaborative study of 228 episodes in a Mediterreanean region Eur J Clin Microbiol Infect Dis 2005 24 411 418 10.1007/s10096-005-1342-6 15928908

[B34] Pintado V Martín-Rabadán P Rivera ML Moreno S Bouza E Visceral leishmaniasis in human immunodeficiency virus (HIV)-infected and non-HIV-infected patients. A comparative study Medicine (Baltimore) 2001 80 54 73 10.1097/00005792-200101000-00006 11204503

[B35] Lopez VR Casado JL Pintado V Decline of visceral leishmaniasis epidemic in HIV infected patients after the introduction of highly antiretroviral therapy Clin Microbiol Infect 2011 7 394 395 10.1046/j.1198-743x.2001.00270.x 11531992

[B36] Del Giudice P Mary-Krause M Pradier C Grabar S Dellamonica P Marty P Gastaut JA Costagliola D Rosenthal E Impact of highly active antiretroviral therapy on the incidence of Visceral leishmaniasis in a French cohort of patients infected with human immunodeficiency virus J Infect Dis 2002 186 1366 1370 10.1086/344325 12402211

[B37] Bernier R Turco SJ Olivier M Tremblay M Activation of human immunodeficiency virus type 1 in monocytoid cells by the protozoan parasite Leishmania donovani J Virol 1995 69 7282 7285 7474154 10.1128/jvi.69.11.7282-7285.1995 PMC189654

[B38] Cacopardo B Nigro L Preiser W Fama A Satariano MI Braner J Celesia BM Weber B Russo R Doerr HW Prolonged Th2 cell activation and increased viral replication in HIV-Leishmania co-infected patients despite treatment Trans R Soc Trop Med Hyg 1996 90 434 435 10.1016/S0035-9203(96)90538-6 8882199

[B39] Barão SC de Fonseca Camargo-Neves VL Resende MR Da Silva LJ Human asymptomatic infection in visceral leishmaniasis: a seroprevalence study in an urban area of low endemicity. Preliminary results Am J Trop Med Hyg 2007 77 1051 1053 18165520

